# Straightforward electrochemical synthesis of a Co_3_O_4_ nanopetal/ZnO nanoplate p–n junction for photoelectrochemical water splitting

**DOI:** 10.1039/d4na00036f

**Published:** 2024-06-18

**Authors:** Khanh Quang Nguyen, Hoang Thai Nguyen, Thach Khac Bui, Tien-Thanh Nguyen, Viet Van Pham

**Affiliations:** a Advanced Materials and Applications Research Group (AMA), HUTECH University 475A Dien Bien Phu Street, Binh Thanh District Ho Chi Minh City 700000 Vietnam pv.viet@hutech.edu.vn; b University of Science Ho Chi Minh City Vietnam; c Vietnam National University Ho Chi Minh City Linh Trung Ward, Thu Duc City Ho Chi Minh City Vietnam; d Institute of Materials Science, Vietnam Academy of Science and Technology 18 Hoang Quoc Viet Hanoi Vietnam

## Abstract

Hydrogen production through photoelectrochemical (PEC) reactions is an innovative and promising approach to producing clean energy. The PEC working electrode of a Co_3_O_4_/ZnO-based p–n heterojunction was prepared by a straightforward electrochemical deposition with different deposition times onto an FTO (Fluorine-doped Tin Oxide) glass substrate. The successful synthesis of the materials was confirmed through analysis using XRD, FTIR, SEM-EDX, DRS, and PL techniques. Mott–Schottky plots and some characterization studies also checked the determination of the formation of the p–n junction. Co_3_O_4_/ZnO/FTO with a Co_3_O_4_ deposition time of 2 minutes exhibited the lowest onset potential of 0.82 V and the lowest overpotential of 470 mV at a current density of 10 mA cm ^−2^. Furthermore, the photo-conversion efficiency of the Co_3_O_4_/ZnO/FTO sample showed 1.4 times higher current density than the ZnO/FTO sample. A mechanism is also proposed to enhance the Co_3_O_4_/ZnO/FTO electrode photo-electrocatalytic activity involved in the water-splitting reaction. The Co_3_O_4_/ZnO/FTO electrode shows significant potential as a promising PEC electrode to produce hydrogen.

## Abstract 2

Hydrogen production through photoelectrochemical (PEC) reactions is an innovative and promising approach to producing clean energy.

## Graphical abstract

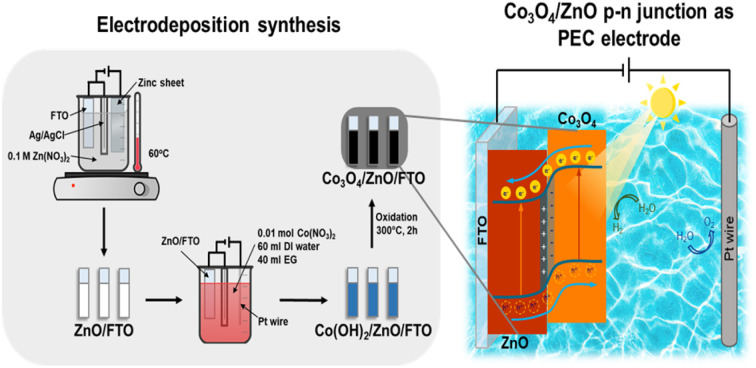

## 1. Introduction

Solar-driven water splitting using semiconductors, including photochemical and photoelectrochemical (PEC) approaches, is receiving significant attention in energy research as a promising solution for clean and renewable energy generation.^1,2^ The research of particulate semiconductor materials for photocatalytic water splitting has emerged as a straightforward and environmentally friendly method for the efficient production of hydrogen.^3^ Efficient photochemical water splitting requires photocatalysts with a band gap higher than the thermodynamic energy requirement of 1.23 eV. Also, the conduction band (CB) needs to be positioned negatively relative to the water reduction potential (H^+^/H_2_; 0 V *vs.* NHE), while the valence band (VB) should be active in a more positive potential region compared to the water oxidation potential (O_2_/H_2_O; 1.23 V *vs.* NHE).^4–6^ Also, the efficiency of photocatalytic water splitting, which converts solar energy into hydrogen, has remained relatively low. For instance, Zhou *et al.*^7^ conducted a study using InGaN/GaN nanowire semiconductors, which achieved one of the highest solar-to-hydrogen efficiencies of 9.2% when using pure water under a xenon lamp with an AM1.5G filter. Integrating electrochemical processes with solar energy in PEC water splitting holds great potential as a highly effective approach for enhancing hydrogen production efficiency.^8^ The primary components of PEC cells consist of a photoactive semiconductor electrode submerged in an appropriate electrolyte and a metal or semiconductor material serving as the counter electrode.^9–11^ However, photoelectrodes in PEC water splitting based on metal oxide semiconductors, *i.e.*, TiO_2_, ZnO, are challenging due to their narrow visible light absorption, unfavorable band positions, low charge mobilities, and less photo-stability, making it challenging to achieve efficient overall performance.^12^ To enhance the separation of photogenerated carriers and expand the absorption of active light, it is advantageous to combine various metal oxides into different heterostructures; therein, the type-II heterojunction is a typical case.^13–15^ In type-II heterojunctions, the movement of holes and electrons occurs in opposite directions, enabling effective separation and transfer of charge carriers at the interface.^16^

Zinc oxide (ZnO ∼3.37 eV) is a semiconductor that exhibits excellent efficiency for PEC due to its CB position (*E*_CB_) at −0.22 V *vs.* NHE, which indicates an opposing alignment relative to the favorable water reduction potential for the hydrogen evolution half-reaction (HER).^17,18^ However, ZnO is responded to UV light excitation, which constitutes only 5% of the total solar energy spectrum.^19^ Besides, the high photo-corrosion of ZnO under UV irradiation in aqueous environments also limits its widespread application.^20^ Meanwhile, cobalt oxide (Co_3_O_4_) possesses two distinct optical band gaps that correspond to the energy state transitions of O^2−^ → Co^3+^ (∼1.4 eV) and O^2−^ → Co^2+^ (∼2.1 eV), leading to the enhancement of electron charge transfer.^21,22^ The nanostructured Co_3_O_4_ films remained stable during chronopotentiometry tests in acidic and alkaline environments.^23,24^ By constructing a Co_3_O_4_ layer onto the ZnO layer, the exposure of ZnO to the aqueous solution can be prevented, thereby mitigating its photo-corrosion. The rapid recombination rate of photoexcited electron–hole pairs is a notable drawback observed in many individual semiconductor materials. As a result, recent studies proposed that the integration of both broad-band gap and narrow-band gap materials or metal-semiconductors could lead to efficient heterojunctions.^25^

The fabrication of ZnO and Co_3_O_4_ p–n junctions has gained attention due to the unique properties of these materials. ZnO, excited by UV light, is employed as the n-type material, while Co_3_O_4_, which can absorb visible light, is the p-type material.^26^ These junctions have shown promising applications in photocatalytic CO_2_ reduction,^27^ degradation of dyes,^28^ and photoelectrochemical water oxidation.^29^ To fabricate a Co_3_O_4_/ZnO p–n heterojunction, ZnO nanomaterials and Co_3_O_4_ nanoparticles were synthesized by many methods, for instance, a combination of the vapor–liquid–solid method at 800 °C and the hydrothermal method for 10 hours at 200 °C.^30^ In another approach, the Co_3_O_4_–ZnO core–shell structure was synthesized by hydrothermally fabricating pristine Co_3_O_4_ nanowires on an FTO glass substrate at 110 °C for 5 hours and the Co_3_O_4_–ZnO film was obtained by annealing in air at 500 °C for 15 minutes using a muffle furnace.^31^ In another study, Markhabayeva *et al.*^32^ employed a combination of spin coating and chemical bath deposition, with two annealing stages at each step to synthesize the ZnO/Co_3_O_4_ on ITO. In contrast to alternative synthesis methods that involve expensive equipment, high temperatures, and long synthesis time, the Co_3_O_4_/ZnO p–n heterojunction was successfully fabricated using the electrodeposition method. This approach not only saves time but also requires a simple equipment setup. The ZnO layer was electrodeposited for 8000 seconds, and the Co(OH)_2_ layer was electrodeposited for 360 seconds. The Co_3_O_4_/ZnO junction was annealed in an ambient environment at 300 °C for 3 hours.^33^

In this study we synthesized a photoelectrode Co_3_O_4_/ZnO/FTO specifically for HER applications. The influence of deposition time on the electrode properties was investigated. The fabricated electrode's characteristics were evaluated using XRD (X-ray diffraction), FTIR (Fourier-transform infrared spectroscopy), SEM-EDX (scanning electron microscopy with energy-dispersive X-ray spectroscopy), DRS (Diffuse Reflectance Spectroscopy), and PL (photoluminescence)—various techniques were used to assess electrochemical properties, including LSV (Linear Sweep Voltammetry) and Mott–Schottky plot. The photoelectrochemical efficiency was also determined by measuring the photocurrent density under simulated solar light conditions. Finally, the mechanism of the hydrogen evolution reaction using the Co_3_O_4_/ZnO/FTO photocathode under simulated solar light illumination is proposed.

## 2. Experimental

### 2.1. Chemicals

Co_3_O_4_/ZnO/FTO materials were fabricated using zinc nitrate (Zn(NO_3_)_2_·6H_2_O, Xilong, China), pure zinc sheet (99.99%), commercial FTO glass (13–15 Ω sq^−1^), cobalt nitrate (Co(NO_3_)_2_·6H_2_O, Xilong, China), deionized (DI) water (Milli-Q, 18 MΩ cm), ethylene glycol (C_2_H_6_O_2_) and acetone (C_3_H_6_O). In addition, sodium sulfate (Na_2_SO_4_) was used in electrochemical measurements.

### 2.2. Synthesis of Co_3_O_4_/ZnO/FTO

A two-step fabrication process was used to synthesize Co_3_O_4_/ZnO/FTO materials. In the first step ZnO was synthesized by electrochemical deposition in a three-electrode system from a 0.1 M zinc nitrate electrolyte solution. FTO glass, a pure zinc sheet (99.99%), and an Ag/AgCl electrode (in a saturated KCl solution) were used as the working electrode (WE), counter electrode (CE), and reference electrode (RE), respectively. FTO glass with a surface resistivity of about 13–15 Ω sq^−1^ was cut into samples of 1.0 cm × 2.0 cm, cleaned with acetone and DI water sequentially in an ultrasonic bath for 20 minutes, then dried. The electrolyte solution was maintained at 60 °C throughout the experiment. The deposition time was 5 minutes with a constant voltage of −1.0 V (*vs.* Ag/AgCl). After deposition, the ZnO/FTO film was washed with DI water and dried in an oven.

**Scheme 1:**
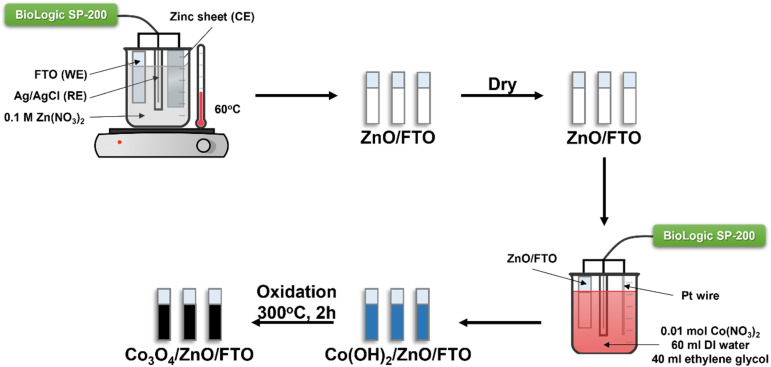
Schematic illustration of the preparation of Co_3_O_4_/ZnO/FTO.

In the second step, the previously prepared ZnO-coated FTO glass was used to continue the deposition of Co(OH)_2_ at room temperature. The electrochemical three-electrode system at this time consisted of ZnO-coated FTO glass, a platinum (Pt) wire, and an Ag/AgCl electrode (in a saturated KCl solution) used as the working electrode, counter electrode, and reference electrode, respectively. The electrolyte solution contained 0.01 mol Co(NO_3_)_2_·6H_2_O, 60 ml DI water, and 40 ml ethylene glycol. The deposition process was carried out at a constant voltage of −1.0 V (*vs.* Ag/AgCl) with the respective survey times of 2, 4, 6, and 8 minutes. Then, Co(OH)_2_/ZnO/FTO was dried and annealed at 300 °C for 2 hours with a constant heating rate of 3 °C min^−1^ to form Co_3_O_4_/ZnO/FTO. The samples are denoted as Co_3_O_4_-2/ZnO/FTO, Co_3_O_4_-4/ZnO/FTO, Co_3_O_4_-6/ZnO/FTO, and Co_3_O_4_-8/ZnO/FTO corresponding to Co_3_O_4_ deposition times of 2, 4, 6, and 8 minutes, respectively. The flowchart of the material synthesis process is described in Scheme 1.

### 2.3. Characterization of materials

The crystal structures of the ZnO/FTO and Co_3_O_4_/ZnO/FTO samples were characterized by XRD using a Bruker D8 Advanced instrument with Cu Kα radiation (*λ* = 1.5406 Å) as the X-ray source, an electron acceleration voltage of 45 kV and a current of 45 mA. The instrument was set to scan from 10° to 80° with a scan rate of 0.02° s^−1^. For FTIR measurements, a JASCO FT/IR-4700 was used to identify the vibrational features of the material's surface functional groups. The sample and KBr mixture were compressed into a round pellet with a diameter of 1 cm and a mass ratio of sample/KBr of 1/300. The compressed pellets were then placed in the instrument for analysis; the IR signal was scanned from wavenumber 4000 cm^−1^ to 400 cm^−1^ with a wavenumber resolution of 1 cm^−1^. The surface morphology of the material was investigated by SEM using a JEOL JSM-IT500. Before observing the SEM images, the electrodes were placed in the measurement chamber with an accelerating voltage of 20 kV. JSM-IT500 was also used for EDX mapping to obtain the elements present in the sample and the weight percentage of each element. The optical properties of the material were determined from the DRS spectrum and the Tauc plot. The DRS method was performed on a JASCO V-770 spectrophotometer in the wavelength range from 300 to 800 nm. The PL spectrum was measured using a Cary Eclipse Fluorescence Spectrophotometer with an excitation wavelength of 300 nm.

Photoelectrochemical measurements, including LSV, photocurrent density, Mott–Schottky, and EIS of the synthesized samples, were performed using a Biologic SP-200 potentiostat and were carried out in a three-electrode system. The working electrode was the investigated material, the counter electrode was a Pt wire, and the Ag/AgCl electrode (in saturated KCl solution) was the reference electrode. All electrochemical experiments were performed at room temperature in 0.1 M Na_2_SO_4_ solution (pH ∼7). LSV measurements were performed at a scan rate of 10 mV s^−1^. The potential was linearly scanned with time while the current was measured and recorded through a reversible hydrogen electrode (V *vs.* RHE) according to eqn (1):

1 *E*_RHE_ = *E*_Ag/AgCl_ + 0059 × pH + *E*^0^_Ag/AgCl_

In this equation, *E*_RHE_ is the potential concerning the reversible hydrogen electrode, *E*_Ag/AgCl_ is the experimentally measured potential, and *E*^0^_Ag/AgCl_ = 0.197 V at 25 °C.

The photocurrent density was determined using a solar simulator device from Abet Technologies. During the measurement, the applied voltage was kept constant at 0.80 V (*vs.* RHE), the light window was turned on and off every 60 seconds per cycle, and the measurement was repeated seven times. Furthermore, Mott–Schottky measurements were performed at a fixed frequency of 100 kHz to obtain the material's conduction band. Electrochemical impedance spectroscopy (EIS) measurements were carried out with a frequency range from 100 kHz to 0.1 Hz and an amplitude of 10 mV.

## 3. Results and discussion

### 3.1. Structural and morphological properties

**Fig. 1:**
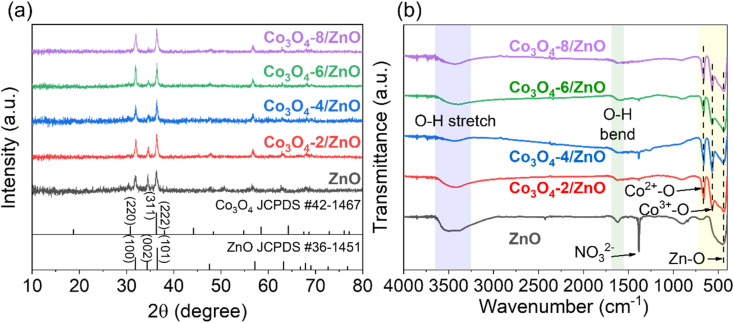
XRD patterns (a) and FTIR spectra (b) of ZnO/FTO and Co_3_O_4_/ZnO/FTO samples.

The synthesized samples' structural and crystalline characteristics were analyzed by XRD and FTIR (Fig. 1). The XRD patterns of ZnO/FTO and Co_3_O_4_/ZnO/FTO samples are shown in Fig. 1a. Accordingly, the characteristic diffraction peaks of ZnO from the standard pattern (JCPDS #36-1451) are observed at diffraction angles (2*θ*) of 31.8°, 34.3°, 36.5°, 47.6°, 57.2°, 63.2°, 67.9°, 69.0°, and 72.7°, corresponding to the (100), (002), (101), (102), (110), (103), (112), (201), and (004) planes, respectively. The XRD results of the synthesized ZnO sample are consistent with the standard pattern, indicating that ZnO has been successfully deposited on the FTO substrate with a hexagonal wurtzite crystal structure.^33^ The prominent diffraction peaks of the ZnO sample corresponding to the (100), (002), and (101) planes have strong intensities, indicating that the formed material has a relatively good crystallinity. In addition, the diffraction peaks in the XRD patterns of Co_3_O_4_/ZnO/FTO samples synthesized with different electrolysis times all match well with the standard diffraction pattern of ZnO (JCPDS #36-1451), indicating that ZnO has been successfully deposited on the FTO substrate in all samples. It was observed that the intensity of the ZnO (101) peak gradually increases, and the intensity of the (002) peak gradually decreases with increasing Co_3_O_4_ deposition time. This was explained by the diffraction peak corresponding to the (311) plane of Co_3_O_4_ in the standard pattern (JCPDS #42-1467), which is the strongest. However, the position of the diffraction peak corresponding to the (311) plane of Co_3_O_4_ was overlapped with that of the ZnO (101) peak. The overlap of the two planes explains the change in the symmetry of the peak at this position, and when the electrolysis time increases, the amount of Co_3_O_4_ on ZnO will increase, leading to increased diffraction intensity. This result partly shows the successful deposition of Co_3_O_4_ on ZnO. However, the XRD patterns of all the samples did not show any clear diffraction peaks of Co_3_O_4_. Therefore, the FTIR method was performed to verify the presence of Co_3_O_4_ (Fig. 1b). In detail, the appearance of the bands at a wavelength of approximately 3440 cm^−1^ and 1600 cm^−1^ can be attributed to the stretching and bending vibrations of the O–H bond, which may be caused by adsorbed water on the material's surface or by atmospheric humidity.^34^ The signal at a wavelength of approximately 455 cm^−1^ is characteristic of the stretching vibration of the Zn–O bond.^35^ At a wavelength of 572 cm^−1^ and 664 cm^−1^, two characteristic peaks appear that correspond to the stretching vibrations of the two bonds: Co(iii)–O when the Co^3+^ ions are in an octahedral coordination state and Co(ii)–O when the Co^2+^ ions are in a tetrahedral coordination state.^36^ These characteristic peaks are present in all Co_3_O_4_/ZnO/FTO samples, demonstrating the successful synthesis of Co_3_O_4_ on the ZnO structure. At the same time, these are also two characteristics of the spinel structure of Co_3_O_4_. This result again confirms the successful synthesis of Co_3_O_4_ on the ZnO/FTO substrate. In addition, the sharp peak at around 1398 cm^−1^ is considered the vibrational signal of the NO_3_^−^ radical. This is likely since both the syntheses of ZnO and Co_3_O_4_ use metal nitrate salts as the electrolyte solution, leading to residual NO_3_^−^ on the material's surface. It is also noted that because the ZnO synthesis process only involves drying without requiring high-temperature annealing, the amount of NO_3_^−^ on the ZnO sample is the highest, leading to the strongest absorption intensity.

**Fig. 2:**
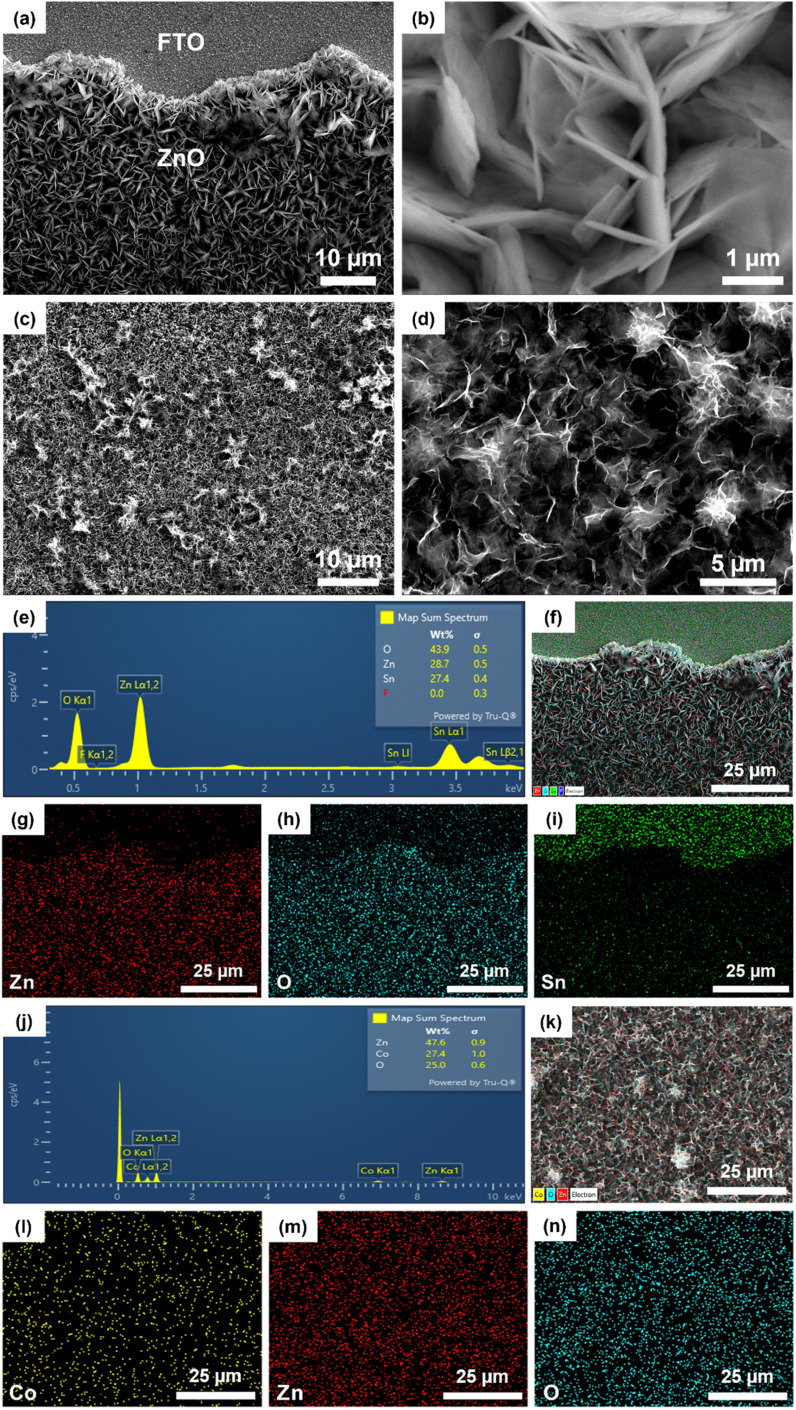
SEM images of the ZnO/FTO sample at magnifications (a) 2000×, (b) 20 000×; SEM images of the Co_3_O_4_/ZnO/FTO sample at magnifications (c) 2000×, (d) 5000×; EDX-mapping of ZnO/FTO (e–i) and Co_3_O_4_/ZnO/FTO (j–n).

The surface morphology of ZnO synthesized by electrochemical deposition on the FTO substrate was observed by SEM. Fig. 2a is a low magnification SEM image (2000×) showing that the surface image of the ZnO film developed uniformly on the FTO substrate. Fig. 2b is a high magnification SEM image (20 000×), indicating that the ZnO obtained has a thin nanoplate structure arranged closely together. SEM also provided the surface morphology of the Co_3_O_4_/ZnO/FTO sample, as in Fig. 2c and d. In detail, the Co_3_O_4_ layer on top has a nanopetal structure that is intertwined and developed uniformly on the ZnO substrate, similar to previous reports on electrochemically deposited Co_3_O_4_.^37^ Thus, based on the SEM images of the samples, it can be concluded that the Co_3_O_4_/ZnO bilayer material with a nanopetal/nanoplate structure on the FTO substrate was successfully synthesized by the electrochemical deposition method with high coverage and uniformity.

In addition, EDX was employed to investigate the elemental composition and potential presence of impurities on the surface of both the ZnO/FTO and Co_3_O_4_/ZnO/FTO samples. The EDX mapping of the ZnO/FTO sample is shown in Fig. 2e–i. The appearance of Zn and O element peaks (Fig. 2e) has proved the successful synthesis of ZnO by electrochemical deposition. In addition, there are also peaks of other elements, such as Sn and F, which are thought to be from the FTO substrate. The mapping images (Fig. 2f–i) show that the elements Zn (red) and O (blue) appear mainly in the ZnO deposition area; at the same time, the uniform distribution of the mapping once again confirms that ZnO was deposited successfully and uniformly on the FTO substrate. Based on EDX mapping of the Co_3_O_4_/ZnO/FTO sample (Fig. 2j–n), the existence of the elements Co, Zn, and O is shown, which implies the successful synthesis of Co_3_O_4_ nanopetals on ZnO/FTO by the electrochemical deposition/annealing method. In addition, no other elements were identified, suggesting that the purity of the synthesized sample is high.

### 3.2. Optical properties

**Fig. 3:**
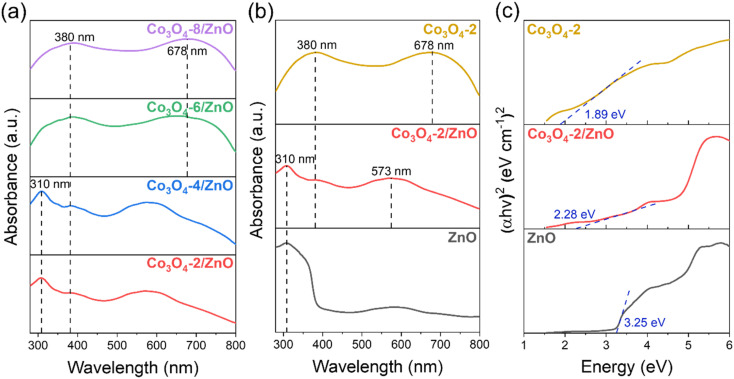
DRS spectra of the synthesized Co_3_O_4_/ZnO samples with different deposition times (a); DRS spectra (b) and Tauc plots (c) of synthesized ZnO, Co_3_O_4_-2, Co_3_O_4_-2/ZnO samples.

To evaluate the optical properties of the materials after fabrication, the DRS spectra of the synthesized Co_3_O_4_/ZnO samples and the Tauc plots are calculated from the DRS spectra of ZnO, Co_3_O_4_-2, and Co_3_O_4_-2/ZnO samples, which are shown in Fig. 3. The Co_3_O_4_/ZnO samples were synthesized with different deposition times (Fig. 3a). For samples with Co_3_O_4_ deposition times from 2 to 4 minutes, clear absorption peaks of ZnO at 310 nm in the UV region are observed. Meanwhile, the DRS spectra of the samples with Co_3_O_4_ deposition times of 6 and 8 minutes show two absorption peaks at 380 nm and 678 nm, respectively, confirming the cubic spinel structure of Co_3_O_4_. The enhancement of the characteristic peaks of Co_3_O_4_ is explained by increasing the deposition time, causing the thickness of Co_3_O_4_ to increase, and losing the typical signal of the ZnO layer. The absorption peak at 380 nm represents the charge transfer of O^2−^ → Co^2+^, and the absorption peak at 678 nm represents the charge transfer of O^2−^ → Co^3+^.^38^ This shows that as the Co_3_O_4_ deposition time increases, the Co_3_O_4_ layer becomes thicker and dominates the material, leading to the inability to record the absorption signal of ZnO. The results from the DRS spectrum also confirmed the successful synthesis of ZnO and Co_3_O_4_ on FTO substrates by electrochemical deposition. In addition, a Co_3_O_4_-2 sample was directly synthesized on the FTO substrate (same process as before with a deposition time of 2 minutes) to compare the material's band gap value before and after the combination. The DRS spectra of the ZnO, Co_3_O_4_-2, and Co_3_O_4_-2/ZnO samples (Fig. 3b) were converted to Tauc plots and are shown in Fig. 3c. According to the results obtained from the Tauc plot in Fig. 3c, the band gaps of ZnO, Co_3_O_4_-2, and Co_3_O_4_-2/ZnO are 3.25 eV, 1.89 eV, and 2.28 eV, respectively. This shows that the combination of the two materials has created a structure with an intermediate band gap value, lying between the original values of ZnO and Co_3_O_4_. This band gap value corresponds to the energy of visible light, so Co_3_O_4_/ZnO photoelectrodes can receive more energy from visible light than ZnO photoelectrodes. The narrowing of the band gap value in the Co_3_O_4_/ZnO material compared to ZnO can be predicted that the photoelectrochemical water splitting ability of the ZnO material can be enhanced when combined with Co_3_O_4_ compared to when in a separate state.

**Fig. 4:**
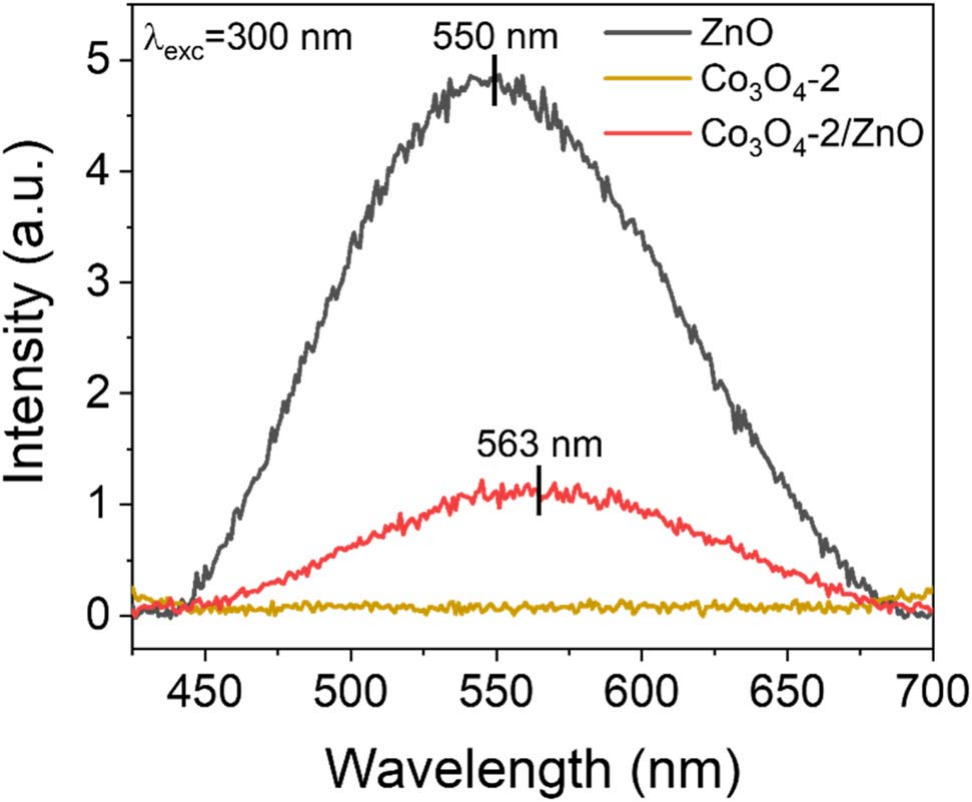
PL spectra of ZnO, Co_3_O_4_-2, and Co_3_O_4_-2/ZnO samples.

PL spectra are used to determine the separation ability and recombination rate between photogenerated electrons and holes in semiconductor material structures. Fig. 4 shows the PL spectra of the synthesized ZnO, Co_3_O_4_-2, and Co_3_O_4_-2/ZnO samples at room temperature. An emission band with broad characteristics was detected at a wavelength of 550 nm in the ZnO layer. This observation can be attributed to defects, specifically singly ionized oxygen vacancies.^39,40^ Upon combining the Co_3_O_4_-2 with the ZnO layer, a noticeable redshift of the emission peak to 563 nm was observed. This suggests that combining Co_3_O_4_-2 and the ZnO layer on FTO can enhance visible light absorption, thereby enabling more efficient PEC water splitting.

The emission intensity in the Co_3_O_4_-2 and Co_3_O_4_-2/ZnO structures is much lower than in pure ZnO. This indicates the better separation and reduced recombination of charge carriers in the Co_3_O_4_-2 structure and the Co_3_O_4_-2/ZnO heterojunction than in ZnO. The difference in emission intensity in the PL spectrum can be attributed to the reduced recombination and extended lifetime of photogenerated electron–hole pairs. This implies that Co_3_O_4_ has the ability to inhibit the recombination of electrons and holes in ZnO, thereby helping to enhance the photocatalytic effects of the material. Moreover, the emission peak at 550 nm of ZnO is typically assigned to the oxygen vacancy defect states of ZnO. Meanwhile, the typical emission peak of Co_3_O_4_ located at 563 nm could be assigned to the band-to-band emission of Co_3_O_4_, which is in agreement with the DRS result analysis in Fig. 3c.

### 3.3. Photoelectrochemical water splitting activity

**Fig. 5:**
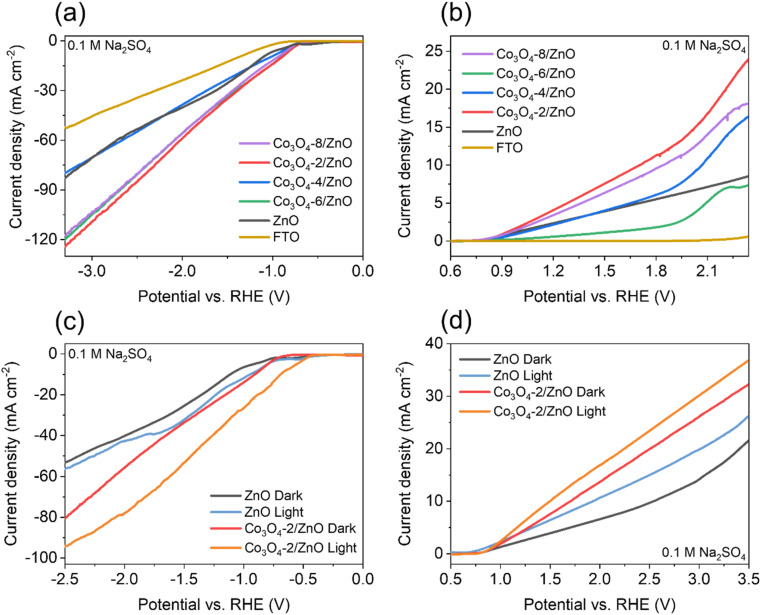
HER (a) and OER (b) curves of FTO, ZnO and Co_3_O_4_/ZnO samples; HER (c) and OER (d) curves of ZnO and Co_3_O_4_-2/ZnO samples under the illuminated and non-illuminated conditions.

The electrochemical water splitting activity of the material is shown by the LSV results (Fig. 5a and b). Co_3_O_4_/ZnO samples with different Co_3_O_4_ deposition times were subjected to LSV (HER) measurements to evaluate the HER activity. The LSV curves were recorded at a scan rate of 10 mV s^−1^ in 0.1 M Na_2_SO_4_ electrolyte solution. In addition, FTO glass and ZnO/FTO (same size) samples were also prepared for LSV measurements for comparison. Fig. 5a shows that the Co_3_O_4_-2/ZnO, Co_3_O_4_-6/ZnO, and Co_3_O_4_-8/ZnO samples have the lowest onset potential, which is close to each other (−0.7 V). However, the Co_3_O_4_-2/ZnO sample has the highest current density (127.7 mA cm^−2^) compared to the other samples. All the material samples used as catalytic electrodes showed a high current density at more negative potentials, and the LSV curves were also linear and straight, indicating that this material is quite stable in the electrochemical water splitting reaction to generate hydrogen.

Similarly, LSV measurements were also performed to evaluate the material's electrochemical water-splitting activity *via* the oxygen evolution reaction (OER). Fig. 5b is a characteristic LSV curve for the OER reaction performed in 0.1 M Na_2_SO_4_ electrolyte recorded at a scan rate of 10 mV s^−1^. The results show that the Co_3_O_4_-2/ZnO sample has the lowest onset potential (0.82 V) and the smallest overpotential of 470 mV at a current density of 10 mA cm^−2^. Based on the two LSV (HER and OER) results, it can be concluded that the Co_3_O_4_-2/ZnO material exhibits the best electrochemical water-splitting activity. Therefore, the optimal deposition time for Co_3_O_4_ on ZnO is 2 minutes. It was also found that the deposition of Co_3_O_4_ on the surface of ZnO can increase the efficiency of the electrochemical water-splitting reaction.

Non-illuminated and illuminated LSV measurements were performed using a solar simulator for ZnO/FTO and Co_3_O_4_-2/ZnO/FTO samples to investigate the photoelectrochemical activity of water splitting. LSV curves were also recorded at a scan rate of 10 mV s^−1^ in 0.1 M Na_2_SO_4_ electrolyte solution. The LSV results for the hydrogen evolution reaction of the materials are shown in Fig. 5c. The results show that both the ZnO and Co_3_O_4_-2/ZnO samples have lower onset and overpotentials when illuminated than when not illuminated. The photocurrent density of the illuminated samples is also higher at the same applied potential. Specifically, the illuminated Co_3_O_4_-2/ZnO sample has an onset potential of −0.5 V and an overpotential of 670 mV, which are lower than the onset potential of −0.7 V and overpotential of 900 mV of the non-illuminated sample. The LSV (OER) curves are shown in Fig. 5d. The LSV results show that, although the onset potential of the illuminated samples shows a slight decrease, when illuminated, both the ZnO and Co_3_O_4_-2/ZnO samples give higher current density and lower overpotential. The illuminated ZnO and Co_3_O_4_-2/ZnO samples have overpotentials of 700 mV and 260 mV, respectively, while under the non-illuminated conditions, the corresponding overpotential values are 1310 mV and 470 mV. From the illuminated LSV results, it can be concluded that light plays an important role in increasing the water splitting efficiency of the material. When light is irradiated onto the material, a higher water splitting current density is observed than in the non-illuminated state (under dark conditions). This suggests that light has excited the electrons in the material, giving them sufficient energy to participate in the water-splitting reaction, thereby increasing the number of electrons participating in the reaction and making the process more efficient.

**Fig. 6:**
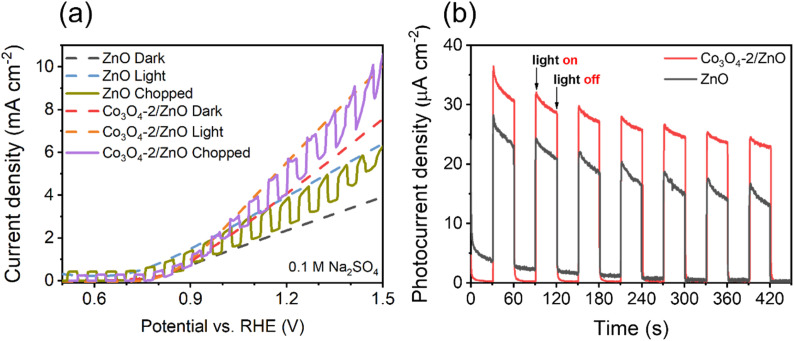
Light-chopped LSV curves (a) and photocurrent density response (b) of ZnO and Co_3_O_4_-2/ZnO samples.

The light-chopped LSV and photocurrent density measurements were performed to determine the material's light-responsive ability, and the results are shown in Fig. 6.

The optimized Co_3_O_4_-2/ZnO and ZnO electrodes were utilized in the chopped LSV experiment to investigate the photoresponse during on–off cycling in the voltage range of 0.5 to 1.5 V *vs.* RHE. The results, shown in Fig. 6a, demonstrate that the chopped LSV curves were consistently similar to the continuous dark and light LSV curves. Additionally, all electrodes exhibited rapid and reproducible photoresponse throughout each on–off cycle. To assess the enhancement of the Co_3_O_4_/ZnO heterojunction compared to ZnO, the steady-state photocurrent was measured at 0.8 V *vs.* RHE. The results in Fig. 6b show that the photocurrent density of the Co_3_O_4_-2/ZnO sample is 1.4 times higher than that of the ZnO sample, from which it can be concluded that the ZnO material has a lower photo-response ability under the action of solar light than the Co_3_O_4_/ZnO material. This may be because ZnO has a large band gap, so it can only absorb energy from the UV region. In contrast, the combined sample may have a smaller band gap, resulting in more light energy being obtained (under the same illumination conditions). At the same time, the light energy conversion efficiency of the ZnO sample also decreases faster than the Co_3_O_4_/ZnO sample after seven cycles, which shows that the combined material has higher stability and photoelectric efficiency than the ZnO material.

Table 1 shows the comparison of different PEC electrodes on the FTO substrate. The electrode fabrication process, involving the electrodeposition of ZnO for 5 minutes and Co(OH)_2_ for 2 minutes, followed by a 3 hours annealing step at 300 °C, offers several advantages. It is a time-saving method that requires simple equipment and operates at a lower temperature than previous studies. Furthermore, the current density at 1.23 V under the same illumination conditions was 5.9 mA cm^−2^. This notable current density is higher when compared to other PEC electrodes based on FTO substrates.^41–45^

**Table 1:** Comparison of PEC electrode performance on FTO substrates under LSV illumination

No.	Material	Synthesis process	Electrolyte	Current density at 1.23 V *vs.* RHE (mA cm^−2^)	Illumination conditions	Ref.
1	BiVO_4_/WO_3_/SnO_2_	SnO_2_ film was initially deposited on the FTO/glass substrate	0.5 M phosphate buffer	3.1	Simulated solar light illumination (100 mW cm^−2^)	41
		WO_3_ was deposited on top of the SnO_2_ film				
		BiVO_4_ nanoparticle film was deposited using the spin coating technique				
2	Fe_2_O_3_/WO_3_ NRs	Deposition of WO_3_ onto FeOOH NRs, then coating on FTO glass and annealing at 450 °C for 4 h	0.1 M Na_2_SO_4_	1.03	Xe 300 W lamp with AM 1.5G	42
3	ZnFe_2_O_4_/α-Fe_2_O_3_	α-Fe_2_O_3_ on FTO: hydrothermal 120 °C for 6 h then annealed at 550 °C for 2 h	1 M NaOH	0.29	AM 1.5G illumination (100 mW cm^−2^)	43
		ZnFe_2_O_4_/α-Fe_2_O_3_ nanorods: depositing ZnO film onto the α-Fe_2_O_3_ of 430 cycles, annealed at 550 °C for 10 h, then washed with 1 M NaOH solution for 10 h to remove ZnO				
4	WO_3_/g-C_3_N_4_	WO_3_/g-C_3_N_4_ heterojunction was prepared by electrophoretic deposition at 10 V then annealed at 350 °C for 2 h	0.5 M Na_2_SO_4_	0.22	Chopped light illumination (AM 1.5G, 100 mW cm^−2^)	44
5	BiVO_4_/ZnCo_2_O_4_	BiVO_4_ nanoworms: growth of BiOI nanoflake structure by electrodeposition and then transformation of BiOI nanoflakes into BiVO_4_ nanoworms by chemical and calcination methods	0.5 M Na_2_SO_4_	1.92	Xe 150 W lamp with AM 1.5G	45
		ZnCo_2_O_4_ nanoparticles: hydrothermal at 20 °C for 12 h then dry 80 °C for 12 h				
6	Co_3_O_4_/ZnO/FTO	Electrochemical deposition ZnO 5 min and Co_3_O_4_ 2 min	0.1 M Na_2_SO_4_	5.9	150 W Xe lamp	This work
		Annealing at 300 °C for 2 h				

The Mott–Schottky method evaluates synthesized material samples' carrier density and electronic conductivity type (p-type or n-type). The type of semiconductor is determined by the slope of the Mott–Schottky plot, with a positive slope indicating an n-type semiconductor and a negative slope indicating a p-type semiconductor. Additionally, Mott–Schottky measurements have been used to determine the materials' conduction band energy level (*E*_CB_) before combination. A Mott–Schottky plot is a graph of the inverse square of the capacitance and the resistance components of the impedance *versus* the applied potential (*C*^−2^*vs. E*) according to eqn (2).2
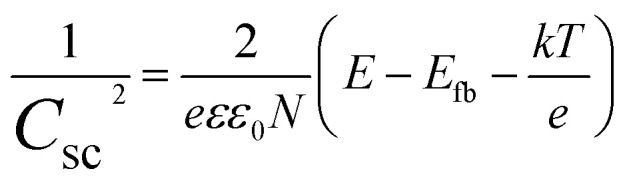
where *C*_sc_ is the capacitance of the space charge region, *ε* the relative dielectric constant of the semiconductor, *ε*_0_ the vacuum permittivity of the free space, *N* is the donor or the acceptor density, *E* the electrode potential, *E*_fb_ the flat band potential, *k* is the Boltzmann constant, and *T* the absolute temperature. The plot of 1/*C*^2^ against the applied voltage will show a tangent that cuts the horizontal axis, the point of intersection will indicate the value of *E*_fb_, also known as the *E*_CB_ of the material.

**Fig. 7:**
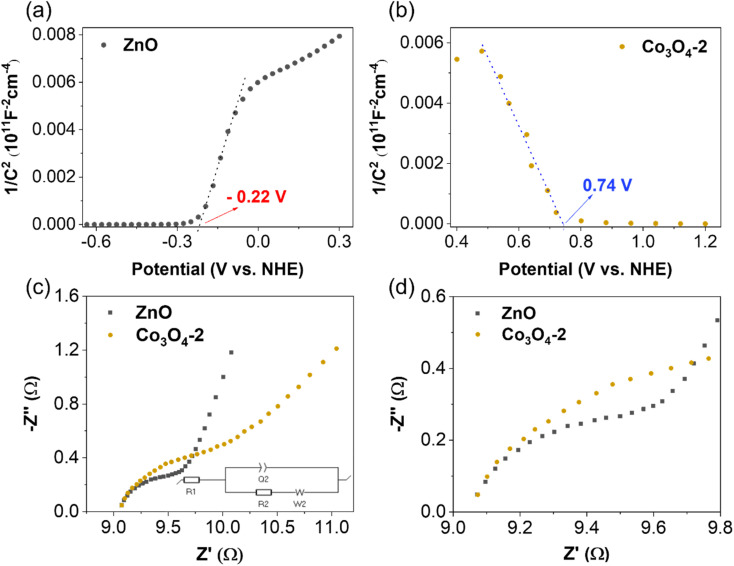
Mott–Schottky plots of (a) ZnO, (b) Co_3_O_4_-2 and (c and d) EIS Nyquist plots.

The Mott–Schottky plot of the ZnO material presented in Fig. 7a shows a positive slope, which indicates that ZnO is an n-type semiconductor. The results show that the conduction band energy level of the ZnO sample is −0.22 V (*vs.* NHE). This result is consistent with the previously reported values of ZnO.^46^ Upon application of external reverse bias to a p-type semiconductor, the depletion region experiences an expansion, consequently leading to an increase in the space-charge capacitance and a subsequent decrease in the value of 1/*C*^2^.^47^ As a result, the negative slope in the Mott–Schottky plot of the Co_3_O_4_ sample (Fig. 7b) provides conclusive evidence for its p-type conductivity, consistent with the presence of majority holes as charge carriers. In addition, the Mott–Schottky results also show that the conduction band energy level of the Co_3_O_4_ material is 0.74 V (*vs.* NHE).

To study the charge-transport behavior existing between the electrode and the electrolyte junction, the data obtained from EIS measurements were analyzed using a simple Randles equivalent circuit (Fig. 7c and d). In this circuit, *R*_1_ is the solution resistance, *R*_2_ (*R*_CT_) is the charge transfer resistance, *Q*_2_ is the double layer capacitance, and *W* is the Warburg impedance. The diameter of the semicircle in the Nyquist plot of EIS is related to the charge transfer resistance and the electron–hole separation efficiency. Therefore, a smaller semicircle diameter indicates a lower charge transfer resistance, *i.e.*, a higher charge transfer efficiency. Based on the EIS results shown in Fig. 7c, the semicircle diameter in the Nyquist plot of the ZnO sample is smaller than that of the Co_3_O_4_ sample. This implies that the electrical resistivity for charge transport for the n-type semiconductor ZnO is lower than that for the p-type semiconductor Co_3_O_4_, and thus promotes the kinetics of surface reactions on ZnO.

**Fig. 8:**
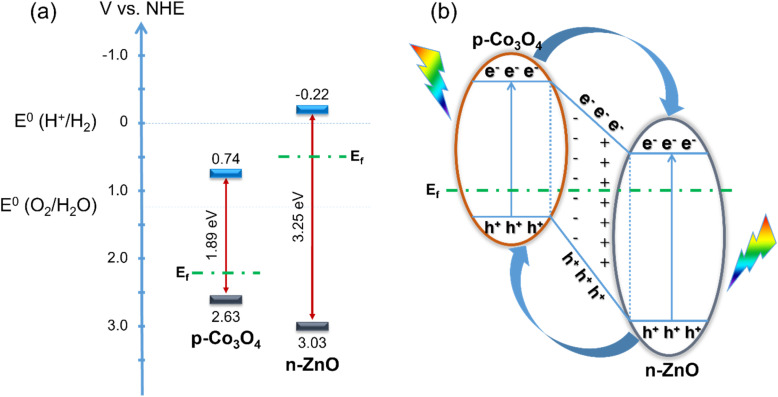
Energy diagram of ZnO and Co_3_O_4_ before combination (a) and p–n junction mechanism between ZnO and Co_3_O_4_ (b).

From the conduction band energy level *E*_CB_, combined with the above band gap values, the energy diagram characteristic of the photoelectrochemical water splitting ability of the material is shown in Fig. 8. The improvement in the photoelectrochemical activity of ZnO for the water splitting reaction by adding Co_3_O_4_ is mainly due to two main reasons. First, the narrow band gap of Co_3_O_4_ helps to reduce the band gap value of the Co_3_O_4_/ZnO material compared to ZnO alone. Second, the p–n junction formed at the interface between the Co_3_O_4_ layer and the ZnO layer increases the concentration of electrons involved in the photoelectrochemical water splitting process.

As illustrated in Fig. 8a, the valence bands (VBs) of Co_3_O_4_ and ZnO are 2.63 V/NHE and 3.03 V/NHE, respectively, the CBs of Co_3_O_4_ and ZnO are 0.74 V/NHE and −0.22 V/NHE, respectively. After combining ZnO, an n-type semiconductor with a band gap of 3.25 eV, with Co_3_O_4_, which can be considered a p-type semiconductor with a band gap of 1.89 eV, the energy levels of Co_3_O_4_ shift up, while those of ZnO shift down until the Fermi levels of Co_3_O_4_ and ZnO reach equilibrium. Therefore, p–n junctions are formed at the interface between ZnO and Co_3_O_4_ and electron transfer occurs (Fig. 8b). When they receive sufficient excitation energy, the excited electrons move to the CB while holes are generated on the VB of both Co_3_O_4_ and ZnO. The electrons on the CB of Co_3_O_4_ can easily move to the CB of ZnO. At the same time, the holes in the VB of ZnO can also move into the VB of Co_3_O_4_, all due to the energy difference. It is also known that most of the charge carriers are holes in the p-type semiconductor Co_3_O_4_ and electrons in the n-type semiconductor ZnO.^48^ Due to the concentration gradient, the holes in Co_3_O_4_ move to ZnO and the electrons in ZnO move to Co_3_O_4_, leaving oppositely charged ions, thus forming an internal electric field with a direction from ZnO to Co_3_O_4_. Electrons tend to move opposite to the electric field, leading to the increasing promotion of electrons on the CB of Co_3_O_4_ to move to the CB of ZnO, helping to increase the electron concentration on ZnO. These electrons directly participate in the water splitting reaction. Therefore, the increasing concentration of electrons in the CB of ZnO increases the photocatalytic activity of the material. In addition, the internal electric field generated at the interface of the two layers of material also plays an important role in slowing down the recombination of the electron–hole pair.^33^ This also significantly enhances the photocatalytic activity of the material.

## 4. Conclusions

The synthesis of the Co_3_O_4_/ZnO/FTO electrode was achieved using a two-step method. The optimal parameters for electrodeposition of ZnO and Co_3_O_4_ were 5 min and 2 min, respectively. This approach was found to be cost-effective and timesaving for synthesizing this type of photochemical electrode. The determination of the formation of the p–n junction was also checked by Mott–Schottky plots and some characterization studies. The Co_3_O_4_-2 and ZnO combination resulted in the formation of p–n junctions, effectively slowing down the recombination of electron–hole pairs. Furthermore, the Co_3_O_4_/ZnO heterojunction electrode expressed a high photo-response ability and the photocurrent density of the Co_3_O_4_-2/ZnO sample is 1.4 times higher than that of the ZnO sample. For the OER, illumination led to overpotentials in the Co_3_O_4_-2/ZnO samples that were 2.7 times lower than those observed in the ZnO samples. Similarly, for the HER, the illuminated Co_3_O_4_-2/ZnO sample, an onset potential of −0.5 V and an overpotential of 670 mV were observed, both of which were lower than the onset potential of −0.7 V and overpotential of 900 mV observed in the non-illuminated sample. In the near future, this combination facilitated efficient visible light absorption, making it a favorable choice for developing highly efficient PEC electrodes for overall water splitting.

## Conflicts of interest

There are no conflicts to declare.
